# Preferential C-nociceptor stimulation facilitates peripheral axon reflex flare, but not secondary mechanical hyperalgesia

**DOI:** 10.3389/fpain.2025.1556429

**Published:** 2025-03-19

**Authors:** Luana Daneffel, Roman Rukwied, Martin Schmelz, Wilhelm Ruppen, Tobias Schneider

**Affiliations:** ^1^ Clinic for Anaesthesia, Intermediate Care, Prehospital Emergency Medicine and Pain Therapy, University Hospital of Basel, Basel, Switzerland; ^2^ Department of Experimental Pain Research, MCTN, Medical Faculty Mannheim, University of Heidelberg, Heidelberg, Germany

**Keywords:** hyperalgesia, C-fibers, flare, chronic pain, electrical nerve stimulation

## Abstract

“Silent” C-nociceptors are crucial for inducing the axon reflex erythema in humans and may also contribute to spinal sensitization such as secondary hyperalgesia. Electrical slow depolarizing stimulation paradigms activate unmyelinated C-fibers [25 ms half-sine (HS) profile] whereas A-fibers are stimulated by 500 µs rectangular (R) pulses. We therefore expect to provoke larger areas of axon-reflex flare (silent nociceptor activation) and secondary hyperalgesia to HS stimuli. We compared axon-reflex erythema and secondary mechanical hyperalgesia areas induced by intracutaneous electrical HS and R stimuli using stimulation intensities that induced pain ratings of 3 and 6 on a numeric rating scale (NRS 0–10) in 24 healthy volunteers. Slowly depolarizing C-fiber stimulation was linked to lower current intensities required to induce pain (NRS 6: HS 3.6 vs. R 9.2 mA, *p* = 0.001) and resulted in larger axon reflex erythema for high stimulus intensities (AUC_Flare_: NRS 6, 320.7 vs. 234.1 cm^2^⋅min, *p* = 0.015; NRS 3, 79.1 vs. 51.0 cm^2^⋅min; *p* = 0.114). Preferential C-fiber stimulation indicated a correlation of axon-reflex erythema with the areas of secondary mechanical hyperalgesia (NRS 6: *r* = 0.21, *p* = 0.036; NRS 3: *r* = 0.48, *p* = 0.0016). In contrast, the mean area of secondary mechanical hyperalgesia did not differ between HS and R [AUC_Hyper_: NRS 6, 1,555 (HS) vs. 1,585 cm^2^⋅min (R), *p* = 0.893; NRS 3, 590 (HS) vs. 449 cm^2^⋅min (R), *p* = 0.212] albeit it developed faster during HS. Our data confirm that silent nociceptors provoke the axon reflex erythema, but their role in secondary hyperalgesia appears to be less crucial.

**Clinical trial number:** NCT0544026

## 1 Introduction

Repetitive activation of skin nociceptors by electrical stimulation provokes pain accompanied by an area of axon reflex erythema and secondary hyperalgesia (1, 2). While the sharp pulses of pain are mediated by thinly myelinated A*δ*-nociceptors, the axon reflex in humans is linked to the activation of C-nociceptors, more precisely of mechano-insensitive “silent” C-nociceptors, whereas polymodal C-nociceptors or A*δ*-fibers are not involved (3–5). Silent nociceptors are of particular clinical interest as they may be sensitized in pathological conditions (6–9) and have been hypothesized to induce spinal sensitization underlying secondary mechanical hyperalgesia and allodynia (10–12). Classic electrical stimulation involves short rectangular pulses that preferentially activate A*δ*-fibers. Recently, there have been attempts to optimize electrical stimulation protocols for C-fibers (13–15) with depolarizing pulses of 25 ms duration being particularly useful for C-fiber activation (16). Thus, we predicted that stimulation with C-fiber-optimized electrical pulses would facilitate the induction of the axon reflex erythema and secondary mechanical hyperalgesia as compared to classic rectangular stimulation.

For this study, we used the established experimental model for pain and secondary hyperalgesia developed by Koppert et al. (1). This human pain model uses repetitive intradermal electrical stimulation to generate stable areas of secondary mechanical hyperalgesia for translational interventional studies (1, 17–20). We compared the classic rectangular pulses to half-sine shaped currents of 25 ms duration, which preferentially activate C-fibers. Slower depolarizing pulses at 4 Hz would have further facilitated the activation of silent nociceptors, but they showed particular adaptation even during a 1 min stimulus (14) and were therefore not suitable for longer-term experiments. Stimulation intensities were set to induce pain levels of either 3 or 6 on an 11-point numeric rating scale. We compared the stimulation profiles in their detection thresholds and current intensities required for pain threshold but also suprathreshold pain levels. We focused on differences in the time course of the induced flare response and in the development of secondary hyperalgesia upon repetitive stimulation using two suprathreshold stimulation intensities that provoked mild (NRS 3) or moderate pain (NRS 6).

## 2 Material and methods

### 2.1 General study design

This study was preregistered at ClinicalTrials.gov (NCT05440266) following approval by the local ethics committee (Ethikkommission Nordwest- und Zentralschweiz, ID 2022-00682). The study was conducted at the University Hospital Basel, Switzerland, after obtaining written informed consent from each participant.

**Figure 1:**
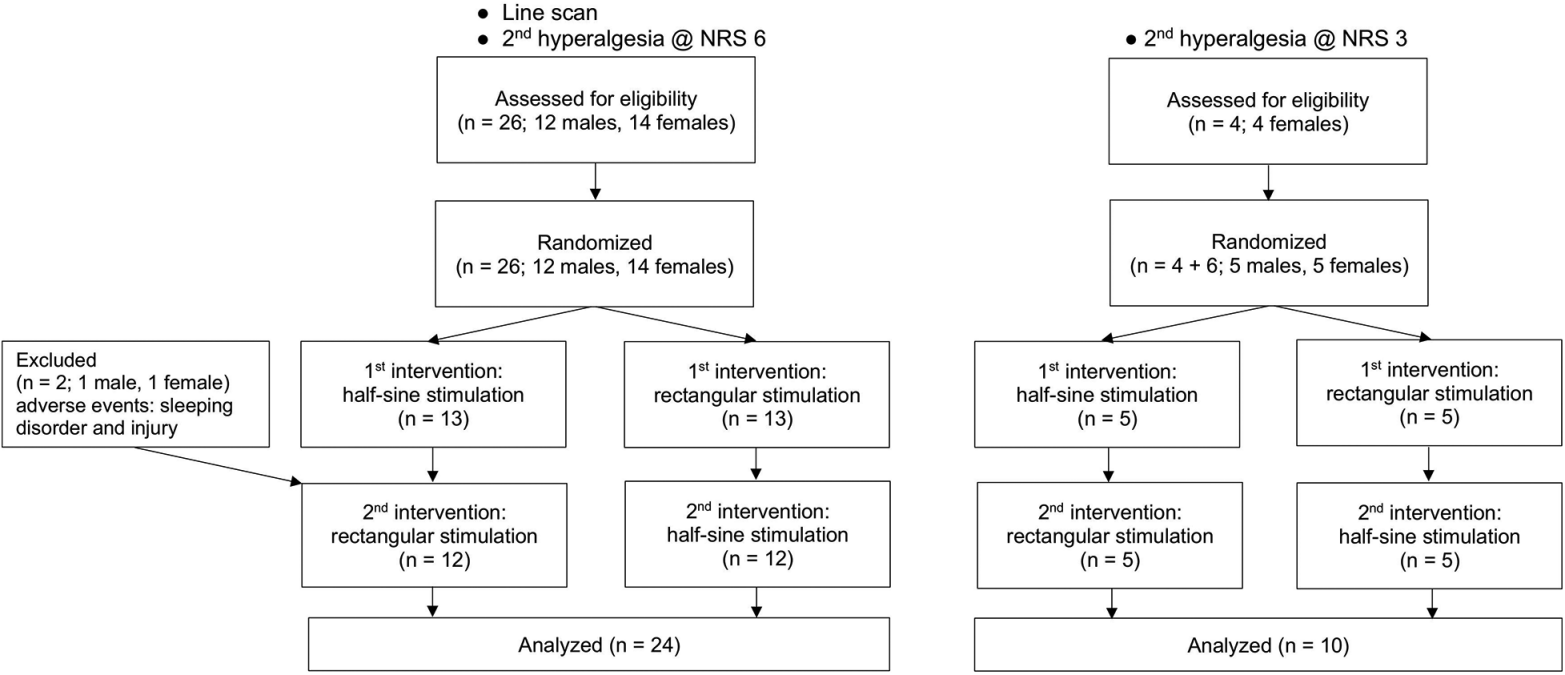
Study flowchart.

**Figure 2:**
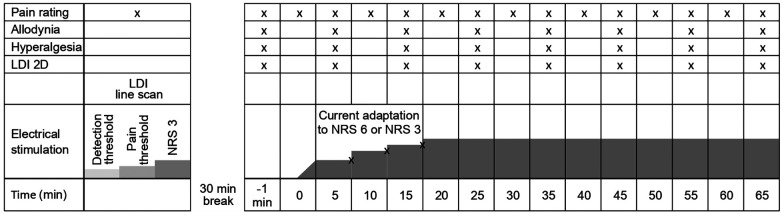
Schematic illustration of the experimental protocol for Experiment II + III showing repeated measurements of pain ratings, allodynia, hyperalgesia, and axon-reflex flare. Adaption of current intensity is possibly made during the first 15 min (according to pain rating). X indicates an assessment of the item at a given time point during the experiment.

This randomized, cross-over, single-blinded experimental proof-of-concept study compared rectangular (R) with half-sine (HS) stimulation at two pain levels (NRS 3 and NRS 6) for the induction of flare and secondary hyperalgesia, in addition to standard threshold assessment. As shown in Figures 1 and 2, each participant began with either the study intervention (half-sine stimulation) or the control intervention (rectangular stimulation). After a minimum interval of 2 weeks, they underwent the alternate intervention. This process was conducted for both protocols, NRS 6 (Experiment I + II) and NRS 3 (Experiment III). Notably, some participants took part in both protocols, while others participated in only one.

### 2.2 Participants

Volunteers were recruited through an advertisement on the homepage of the University of Basel, and inclusion occurred on a “first come, first served” basis with a balanced sex distribution (50% male, 50% female). The inclusion criteria included healthy [American Society of Anesthesiologists Physical Status (ASA) I–II] adults (aged 18–65 years) with a body mass index (BMI) of 18.5–25 kg/m^2^. The exclusion criteria included regular intake of pain-modifying drugs (i.e., analgesics, opioids, antihistamines, calcium and potassium channel blockers, antidepressants, and corticosteroids); presence of neuropathy, chronic pain, neuromuscular disease, dermatological disease (i.e., atopic dermatitis), and psychiatric disease; or pregnancy/lactation. Female participants of childbearing age were tested for pregnancy prior to each intervention and received information concerning contraception in the participant information.

The experimental setup, including the assessment of pain according to the numeric rating scale (NRS; 0 = no pain to 10 = worst pain imaginable), sensory testing (allodynia, hyperalgesia), and the use of the laser Doppler imaging (LDI), was explained to each participant prior to the first intervention. The participants received financial compensation.

### 2.3 Pain model

After providing cold analgesia using an ice pack attached to the forearm skin sites designated for microdialysis, two microdialysis catheters (outer diameter, 0.2 mm) were inserted intracutaneously and in parallel into the participant's non-dominant volar forearm using 25 G cannulas and at a length of approximately 10 mm, separated from each other by 5 mm, a configuration used for electrically evoked secondary hyperalgesia since 2001 (1). The microdialysis catheters were each equipped with an internal stainless-steel wire, connected to a constant current stimulator (Digitimer DS7A or DS5, Digitimer Ltd., Welwyn Garden City, UK). The catheters were perfused with 0.9% saline at a continuous flow of 0.4 µl/min guaranteed by a syringe pump (CMA 402, Harvard Bioscience, Holliston, MA, USA).

For the study intervention, electrical half-sine pulses of 25 ms duration in alternating polarity were applied at 2 Hz by a constant current stimulator (Digitimer DS5, Digitimer Ltd., Welwyn Garden City, UK) controlled by DAPSYS 8 (https://www.dapsys.net).

In a separate session, the established stimulation with rectangular pulses of 500 µs duration in alternating polarity was applied at a 2 Hz frequency by a constant current stimulator (Digitimer DS7A, Digitimer Ltd., Welwyn Garden City, UK) connected to a pulse generator (PG1, Rimkus Medizintechnik, Parsdorf, Germany) and served as the control.

#### 2.3.1 Threshold evaluation

Electrical stimulation through the intracutaneous electrodes started at 0.1 mA, and the intensity was increased in 0.1 mA steps per 3 s. The subjects were asked to indicate when they first felt the stimulation (detection threshold). Stimulus intensity was then further increased until the subjects reported the first pain sensation (pain threshold) and, finally, until they reported a targeted pain level of NRS 3 (suprathreshold pain). At least six pulses were applied for each threshold, and the intensities (mA) were recorded. The participants were blinded to the milliampere values applied.

**Figure 3:**
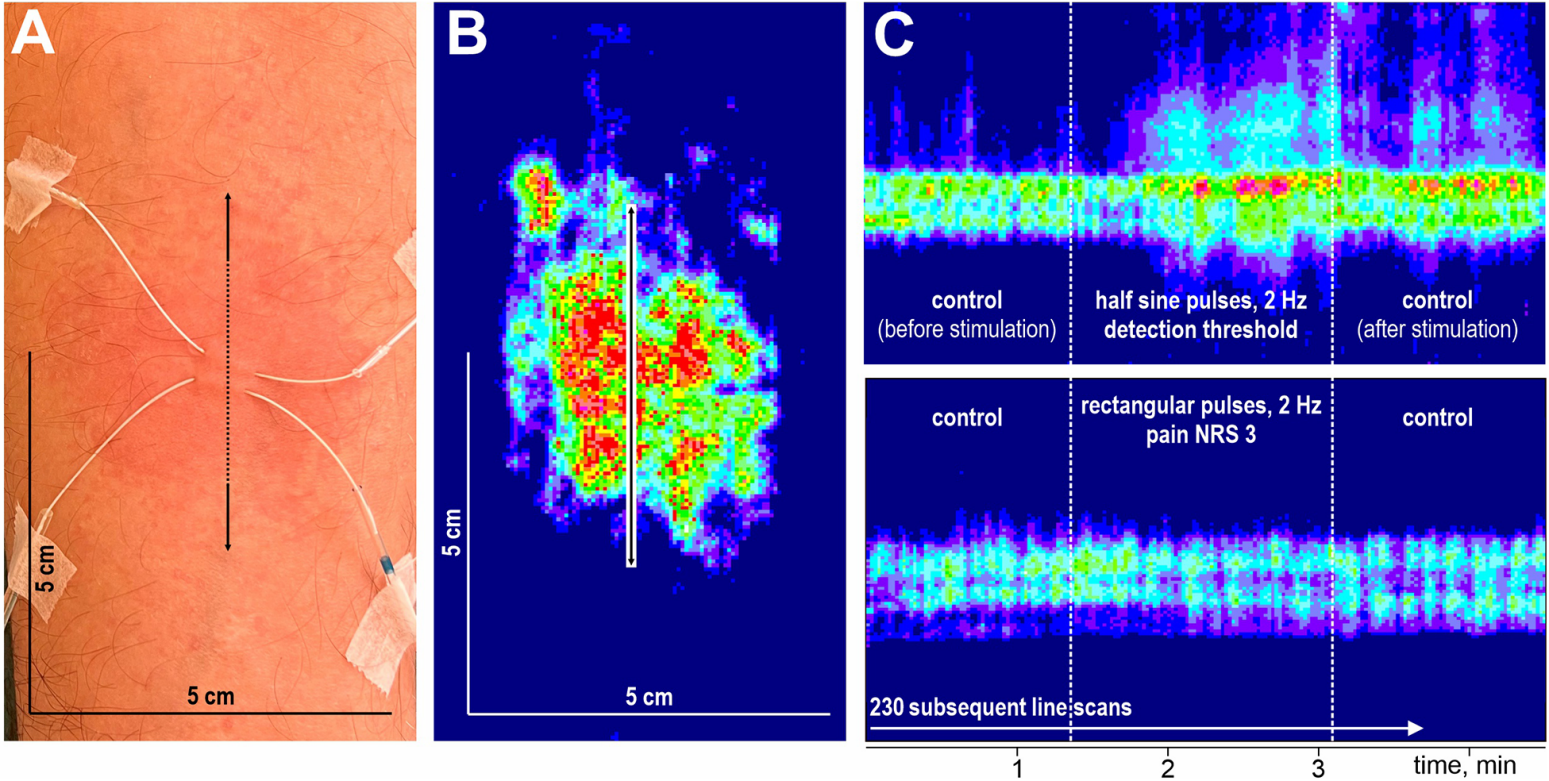
Schematic overview of stimulation and flare recording. Microdialysis catheter at the volar forearm, detection area of 5 × 5 cm, the arrow indicates laser Doppler scan direction **(A)**. Corresponding laser Doppler image after flux correction **(B)**. Laser Doppler image of flare response at high temporal resolution during half-sine and rectangular stimulation **(C)**.

After a 15 min break, electrical pulses at the detection threshold, pain threshold, and pain level NRS 3 were applied for 106 s at 2 Hz, and the instant flare reaction was recorded by laser Doppler line scans across the stimulation site. The experimental setup (A), the electrically evoked axon reflex erythema with laser Doppler line scan orientation (B), and laser Doppler line scan sequences recorded upon half-sine (C, top) and rectangular (C, bottom) stimuli are depicted in Figure 3.

#### 2.3.2 Axon reflex with high temporal resolution (“line scan”)

Changes in superficial blood flow were assessed by laser Doppler imaging (LDI, Moor Instruments Ltd., Devon, UK) at high temporal resolution (1.18 s per scanned line) using repetitive line scans of 5 cm length oriented across the stimulation site (Figure 3B). After a baseline of 70 lines, the electrodes were stimulated for 106 s (90 lines) followed by a control period of 70 lines without stimulation (Figure 3C). The spatial extent of the induced axon reflex was analyzed offline (Research Version 5.3, 2009, Moor Instruments Ltd., Axminster, UK). Pixels were defined as flare when their flux values exceeded baseline level plus twofold standard deviation, and the spatial extent was assessed by the length measuring tool of the moorLDI software as performed previously (16, 21), according to Chizh et al. (22) and Geber et al. (23). The maximum length before, during, and after stimulation were analyzed (cf. Supplementary Figures S3 and S4). To evaluate the temporal aspect, we identified the stimulation pulse at which blood flow exceeded 125% of the length at baseline.

#### 2.3.3 Secondary mechanical hyperalgesia

After a 30 min stimulation break following the final line scan recorded after threshold evaluation, baseline measurements of hyperalgesia, allodynia, and spatial extent of the axon reflex erythema were assessed. Thereafter, repetitive electrical stimulation at 2 Hz commenced according to the participant's pain rating of NRS 3 (35 min assessment) or NRS 6 (65 min assessment). After 5, 10, and 15 min, stimulation intensity was readjusted to compensate for habituation and kept thereafter constant for the remaining time period of the protocol (Figure 2). During continuous electrical stimulation, the extent of hyperalgesia and allodynia was assessed every 10 min (six times for NRS 6 and three times for NRS 3). Pinprick hyperalgesia was assessed using a 256 mN von Frey filament (MRC Systems GmbH, Heidelberg, Germany), and allodynia was determined using a dry cotton swab (MediSet, IVF Hartmann, Neuhausen, Switzerland). In both cases, the test stimuli were approaching the site of electrical stimulation in 0.5 cm increments from four orthogonally oriented lines from proximal, distal, lateral, and medial sites. Starting points were well outside the expected hyperalgesic area, 12 cm from the site of electrical stimulation for distal and proximal measurements and 6 cm for lateral and medial measurements. Participants were instructed to report when the sensation either increased in pain from the pinprick (hyperalgesia) or an unpleasant “rougher” sensation occurred from the cotton swab (allodynia). Assuming the test area to be elliptical, the area of hyperalgesia and allodynia was calculated by using the formula ¼ *π D*·*d* (where *D* is the diameter in the sagittal and *d* is the diameter in the transverse axis of the forearm).

#### 2.3.4 Spatial extent of axon reflex

Laser Doppler scans of 11.6 × 5.7 cm were taken at 10 min intervals before assessment of the hyperalgesia and analyzed offline for flare size development. As mentioned above, pixels for which flux exceeded baseline level plus twofold standard deviation were encountered to the axon reflex flare, and their area was assessed by dedicated software (Research Version 5.3, 2009, Moor Instruments Ltd., Axminster, UK) (cf. Supplementary Figures S5 and S6).

### 2.4 Endpoints

The primary endpoint was the secondary hyperalgesia measured as the area under the curve (AUC) during constant current simulation for 65 min at pain level NRS 6 (AUC_Hyper_). The main comparison was the difference of the AUC during 25 ms half-sine stimulation vs. 500 µs rectangular stimulation.

Secondary endpoints were pain rating (NRS), axon-reflex flare, and extent of allodynia during stimulation at pain levels NRS 6 and NRS 3 (as AUCs) between the stimulation profiles. In addition, the development of secondary hyperalgesia during stimulation was compared between the two profiles. For both pain levels, we investigated correlations between the area of hyperalgesia and the corresponding flare response. The different current intensities (in mA) for the detection threshold, pain threshold, and pain levels NRS 6 and NRS 3 as well as after adaption were compared between the two profiles. Moreover, the instant flare reaction (laser Doppler line scan signal) generated during threshold testing and the axon-reflex flare upon ongoing stimulation were compared between the stimulation profiles.

### 2.5 Statistical methods and data presentation

#### 2.5.1 Sample size

The sample size was estimated with the aim of showing a significant difference between the area of secondary hyperalgesia (AUC_Hyper_) during stimulation with the two stimulation profiles. The significance level was chosen to be *α* = 5%, while the power was chosen to be at least (1 – *β*) = 90% when the difference of mean AUC_HyperHS_ and AUC_HyperR_ was *θ* = 20% based on the experience of development of hyperalgesia during prior trials using this model (24). Assuming a proportional change of mean AUC_Hyper_ *θ* = −20% and a dropout rate of 10%, a total of 23 participants should have been recruited to achieve a total of *n* = 20 evaluable participants. No power calculation was performed for the 35 min assessments at pain level NRS 3 due to lack of preliminary data, and therefore only 11 participants were recruited to achieve a total of *n* = 10 evaluable participants.

#### 2.5.2 Statistical analysis

We quantified the current intensities, differences in skin blood flow (line scan extension), and applied pulses (until a flare reaction occurred) for the two stimulation protocols and performed a paired two-sample *t*-test. No adjustments for *P*-values were made.

Pain scores during continuous electrical stimulation and current adaptation in the first 15 min were analyzed by paired two-sample *t*-test as well. To compare the areas of hyperalgesia, allodynia, and axon-reflex flare over time (each as a dependent variable), we calculated the AUC by using the trapezoid formula and analyzed these data using analysis of variance (ANOVA) including current type and time point of measurement as inner subject factors. Bonferroni *post hoc* testing was performed. A repeated-measures ANOVA was used to compare the area of hyperalgesia between the stimulation profiles at single time points. Normal distribution was checked using a Kolmogorov–Smirnov test. Only detection thresholds and allodynia were not normally distributed, and thus additional non-parametric Wilcoxon rank tests were performed confirming the parametric tests. We report the results of *t*-tests and ANOVA as predefined in our study protocol for all parameters based on the robustness of the repeated measures ANOVA against violation of normality (25, 26).

We used a linear correlation model reporting Pearson’s *r* for correlation analysis between hyperalgesia and flare response.

Statistical analyses were performed with SPSS (IBM SPSS Statistics, 2021; New York, NY, USA), and graphical content was designed with GraphPad Prism (Version 9, 2020; San Diego, CA, USA).

#### 2.5.3 Data presentation

Participant characteristics are shown as numbers (proportions), medians with interquartile ranges (IQR), and mean ± SD as appropriate (Table 1). All outcome parameters of primary and secondary endpoints are presented as means ± SD or absolute numbers in descriptive reports.

**Table 1:** Baseline characteristics.

Participant characteristics	Experiments I + II (NRS 6; 65 min)	Experiment III (NRS 3; 35 min)
No. of participants	24	10
Age	24.5 (4.75)	25.5 (4.25)
Male	11 (45%)	5 (50%)
Female	13 (55%)	5 (50%)
BMI (kg/m^2^)	22.3 ± 1.5 kg/m^2^	21.8 ± 1.6 kg/m^2^
Ethnicity		
Caucasian	20 (84%)	10 (100%)
East Asian	1 (4%)	
South Asian	2 (8%)	
Black African	1 (4%)	

Data are shown as median with interquartile range (age) and mean with SD (BMI).

BMI, body mass index.

## 3 Results

### 3.1 Baseline characteristics

The participants (*n* = 26: 22 Caucasian, 2 South Asian, 1 Black African, 1 East Asian) were recruited in a balanced sex distribution [14 women (54%), 12 men (46%)] from May to November 2022. The participants ranged in age from 21 to 55 years (median = 24.5; IQR = 4.75), and the mean BMI was 22.3 ± 1.5 kg/m^2^. Two participants dropped out: one was not able to follow the instructions during the intervention due to psychological issues, and one had an accident that impaired skin blood flow and nociception before the second session. Only completed measurements were analyzed (Figure 1).

### 3.2 Dose response and immediate flare induction

**Figure 4:**
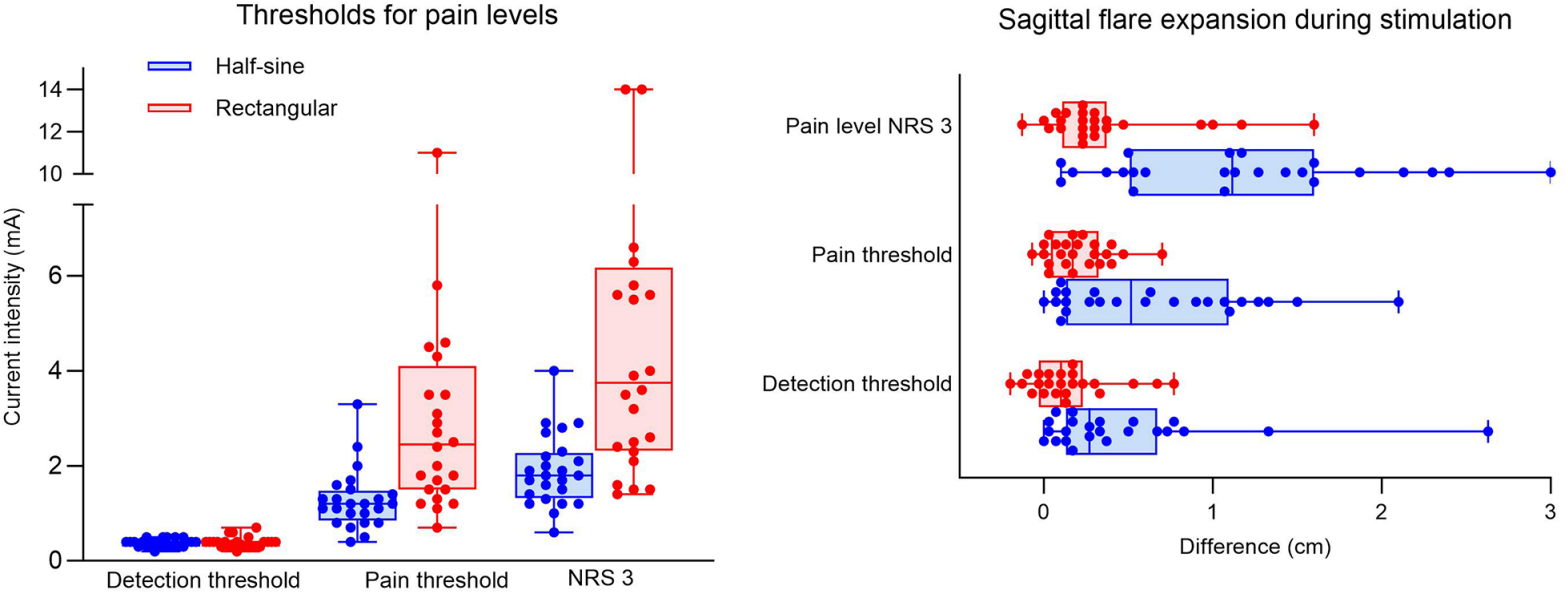
Experiment I. Box plots showing intensity values (mA) for dose response at detection threshold, pain threshold (NRS 1), and pain level (NRS 3) with half-sine or rectangular-shaped stimulation for all participants (dots) **(A)**. Sagittal expansion (in cm) of flare during half-sine or rectangular stimulation at the three different (pain) levels for all participants (dots) **(B)**. NRS, numeric rating scale.

The mean current intensities to reach the detection threshold were similar for both stimulation profiles [0.39 ± 0.11 mA (R) vs. 0.37 ± 0.09 mA (HS)]. However, approximately 2.5 times higher current intensities were required to reach the pain threshold (3.12 ± 2.43 mA vs. 1.28 ± 0.62 mA; *p* = 0.001) and a pain level of NRS 3 (4.88 ± 3.56 mA vs. 1.90 ± 0.75 mA; *p* = 0.001) for R stimulation vs. HS stimulation (Figure 4).

At the detection threshold, the mean sagittal expansion of the axon reflex during both interventions was 0.15 ± 0.24 cm (R) and 0.46 ± 0.58 cm (HS), *p* = 0.023. Only three participants during R and six participants during HS developed a persistent flare reaction at the detection threshold. Stimulation at the pain threshold elicited a flare reaction in 3 participants during R and 12 participants during HS stimulation, and the extension differed significantly between the groups [0.21 ± 0.18 cm (R) vs. 0.64 ± 0.57 cm (HS), *p* = 0.001]. At pain level NRS 3, most participants (20 of 24) developed a flare reaction during HS stimulation, whereas during R stimulation only 7 had a flare reaction with a significant difference in spatial extension of 1.17 ± 0.79 cm during HS vs. 0.37 ± 0.41 cm during R stimulation (*p* = 0.001) (Figure 4).

### 3.3 Pain, hyperalgesia, allodynia, and flare

**Figure 5:**
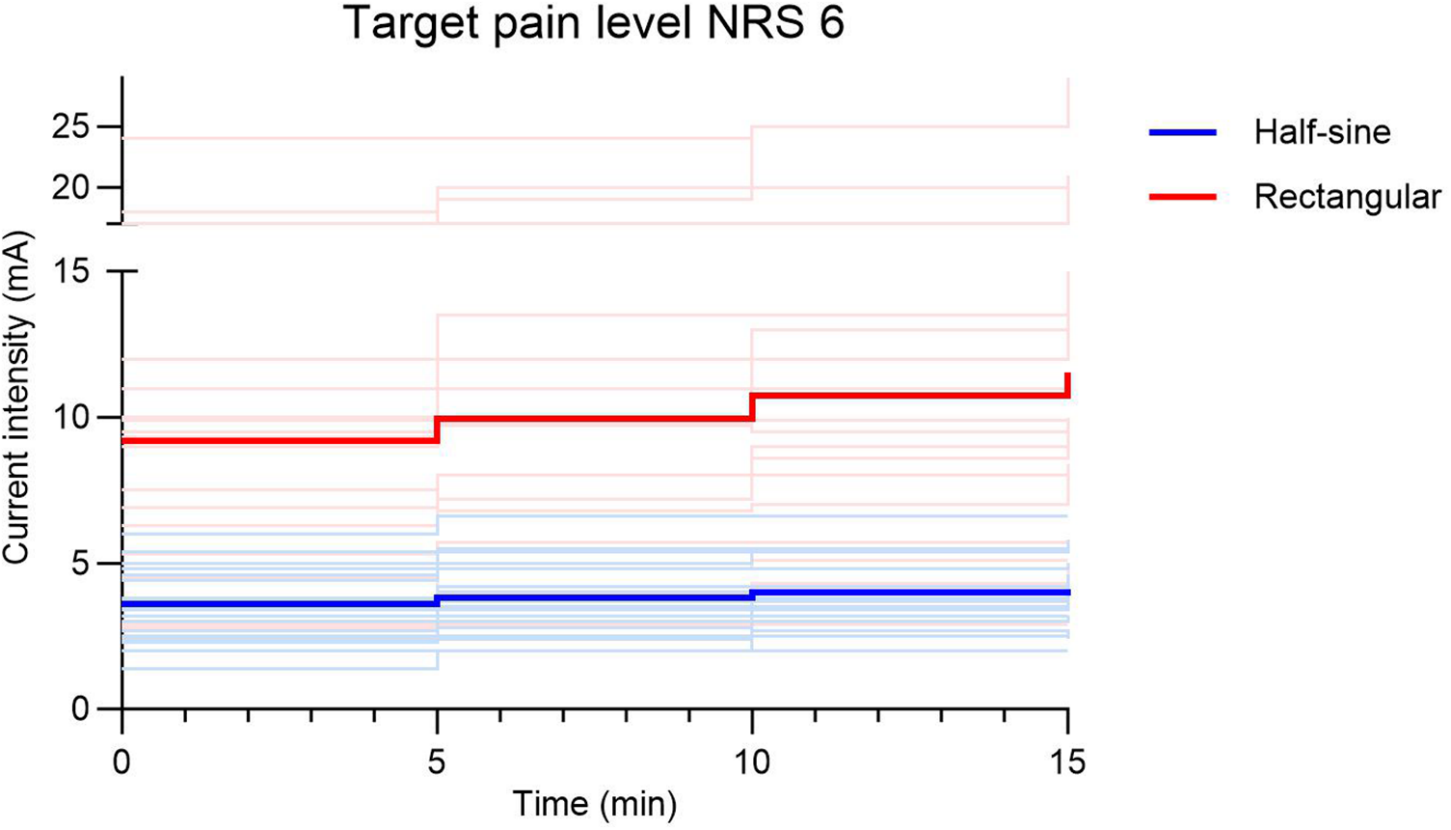
Adaptation of current intensity (mA) during the first 15 min of stimulation to target pain level NRS 6 shown as bright lines for individuals and bold lines as the average of the respective stimulation group (half-sine or rectangular). NRS, numeric rating scale.

**Figure 6:**
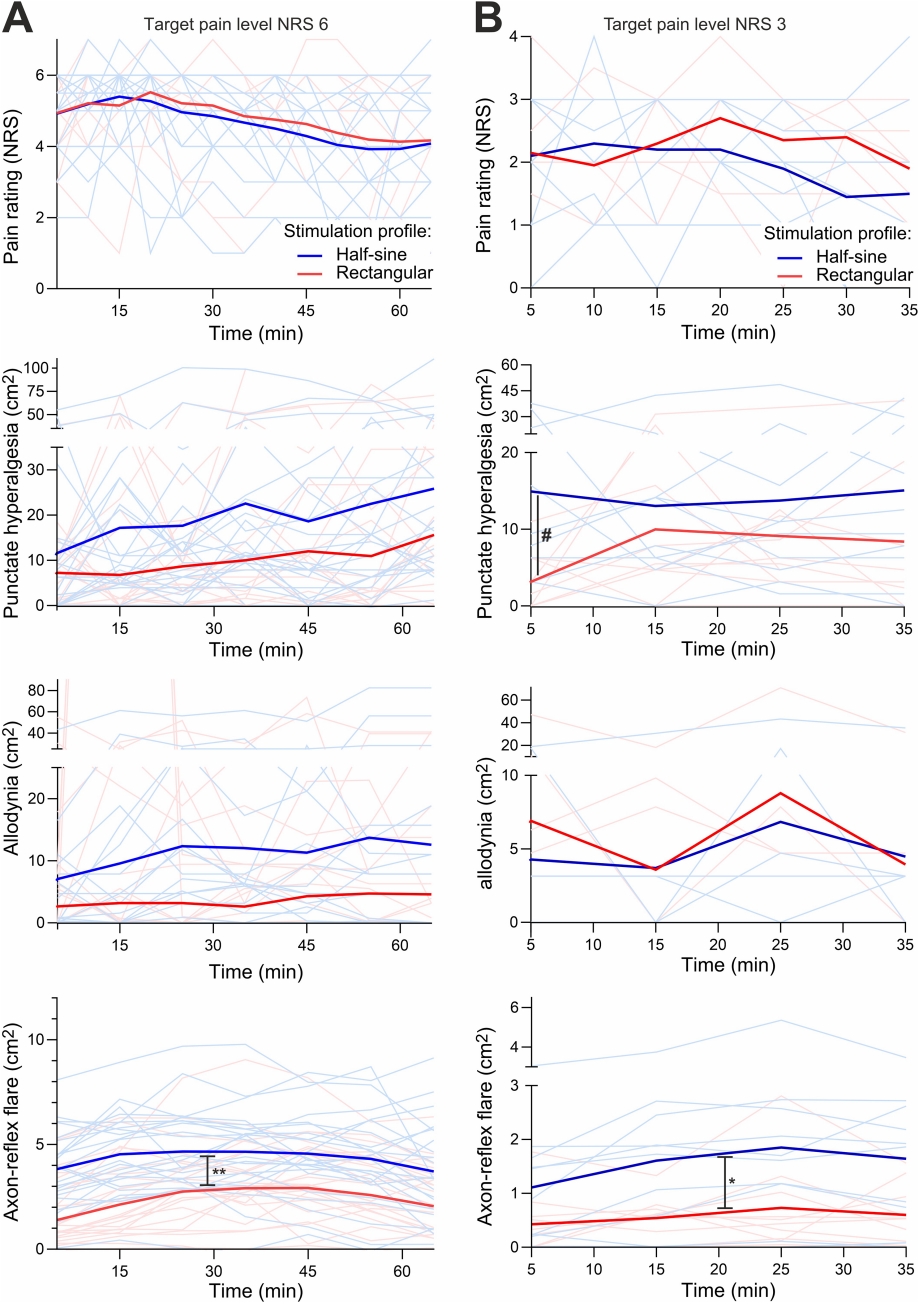
Experiment II + III. The course of pain level (NRS), areas of secondary hyperalgesia, allodynia, and flare (cm^2^) over time shown as bright lines for individuals and bold lines as the average of the respective stimulation group (half-sine or rectangular) at target pain level NRS 6 **(A)** and at NRS 3 **(B)**. Allodynia at **B** shows less bright lines, as some lines run at level 0 because no allodynia occurred. NRS, numeric rating scale. # marks the significant difference of hyperalgesia after 5 min; * and ** mark the significant difference of axon-reflex flare for NRS 3 and 6 over time, respectively.

To target a pain level of NRS 6, current intensities were 2.5–3 times higher during R vs. HS stimulation (9.2 ± 5.34 mA vs. 3.6 ± 1.17 mA, respectively; *p* = 0.001). Additionally, the increase of current to compensate for habituation during the first 15 min of stimulation was 3.5–6.5 times higher during R (0.75–0.78 mA) vs. HS stimulation (0.12–0.21 mA) (Figure 5). The mean average pain score (NRS) over time was 4.8 ± 0.4 during R stimulation and 4.6 ± 0.5 during HS stimulation (*p* = 0.19) (Figure 6A). To induce pain levels of NRS 3, current intensities were 2.5 times higher during R vs. HS stimulation (3.8 ± 1.3 mA vs. 1.5 ± 0.5 mA, respectively; *p* < 0.01). To compensate for initial habituation during the first 15 min of measurement, current intensities had to be increased by an average of 0.5 mA during R and 0.2 mA during HS stimulation. During R stimulation, the mean of the average pain score (NRS) over time was 2.3 ± 0.3 vs. 2.0 ± 0.4 during HS stimulation (*p* = 0.127) (Figure 6B).

#### 3.3.1 Assessment of hyperalgesia and allodynia

For the higher stimulation intensity (NRS 6), AUC_Hyper_ (at 65 min) did not differ [1,584.8 ± 1,233.1 cm^2^·min (R) vs. 1,555.21 ± 1,225.8 cm^2^·min (HS), *p* = 0.893] between the two stimulation profiles. The mean area of hyperalgesia over all time points of assessment increased continuously in both intervention groups and reached a maximum after 35 min [26.8 ± 20.9 cm^2^ (R) vs. 26.4 ± 21.9 cm^2^ (HS)]. The maximum growth of the area occurred during the first five minutes of stimulation and was greater with HS stimulation (+190% vs. 142%) with an absolute increase of 9.5 cm^2^ (HS) compared to 7.1 cm^2^ (R) (*p* = 0.57) (Figure 6A). During HS stimulation, 16 out of 24 participants developed hyperalgesia, whereas it occurred in 14 participants during R stimulation.

Regarding AUC_Allo_, HS stimulation tended to produce a larger area of allodynia but without reaching statistical significance [549.2 ± 814.5 cm^2^⋅min (R) vs. 681.3 ± 910.4 cm^2^⋅min (HS); *p* = 0.206, Figure 6A]. However, there was a great within- and inter-participant variability regarding allodynia.

For the lower stimulus intensity (NRS 3), AUC_Hyper_ over 35 min was not significantly larger during HS (590.2 ± 464.2 cm^2^⋅min) vs. during R stimulation (448.7 ± 406.1 cm^2^⋅min; *p* = 0.212). The maximum spread of secondary hyperalgesia was achieved during the first 5 min of HS stimulation (area after 5 min during R 8.3 ± 6.1 cm^2^ vs. HS stimulation 19.0 ± 17.5 cm^2^; *p* = 0.042). The maximum area of hyperalgesia was slower to develop during R stimulation, with maximum extension after 15 min (15.5 cm^2^) (Figure 6B). The absolute increase (during the first 5 min of stimulation) of the area of secondary hyperalgesia was also greater with HS (14.9 cm^2^) vs. R stimulation (2.7 cm^2^; *p* = 0.005).

AUC_Allo_ at 35 min did not differ between the two stimulation types (227.9 ± 364.4 cm^2^⋅min vs. 218.7 ± 429.6 cm^2^⋅min for HS vs. R stimulation, respectively; *p* = 0.879; Figure 6B). Allodynia developed in 4 of 10 participants and in 6 of 10 participants during R and HS stimulation, respectively.

#### 3.3.2 Assessment of flare

For the higher stimulation intensity (NRS 6), AUC_Flare_ at 65 min was 234.1 ± 140.8 cm^2^⋅min during R and 320.7 ± 108.6 cm^2^⋅min during HS stimulation (*p* = 0.015). The maximum extent of flare was reached by most participants at 35 min [8 of 24 (R) vs. 16 of 24 (HS)]. The greatest increase in area was measured during the first 5 min of stimulation, with 1.79 cm^2^ (R) and 3.9 cm^2^ (HS) representing a 37% vs. 78% respective increase compared to prior size (Figure 6A).

For the lower stimulus intensity (NRS 3), the AUC_Flare_ at 35 min was 51.0 ± 27.0 cm^2^⋅min during R vs. 79.1 ± 38.9 cm^2^⋅min during HS stimulation (*p* = 0.114). Both stimulation types achieved maximum expansion after 25 min (1.7 ± 0.9 cm^2^ during R vs. 2.6 ± 1.5 cm^2^ during HS stimulation; *p* = 0.051). The greatest area increase was measured during the first 5 min of stimulation with 0.4 cm^2^ during R and 1.1 cm^2^ during HS stimulation (*p* = 0.027; Figure 6B).

### 3.4 Correlations between areas of flare and secondary hyperalgesia

**Figure 7:**
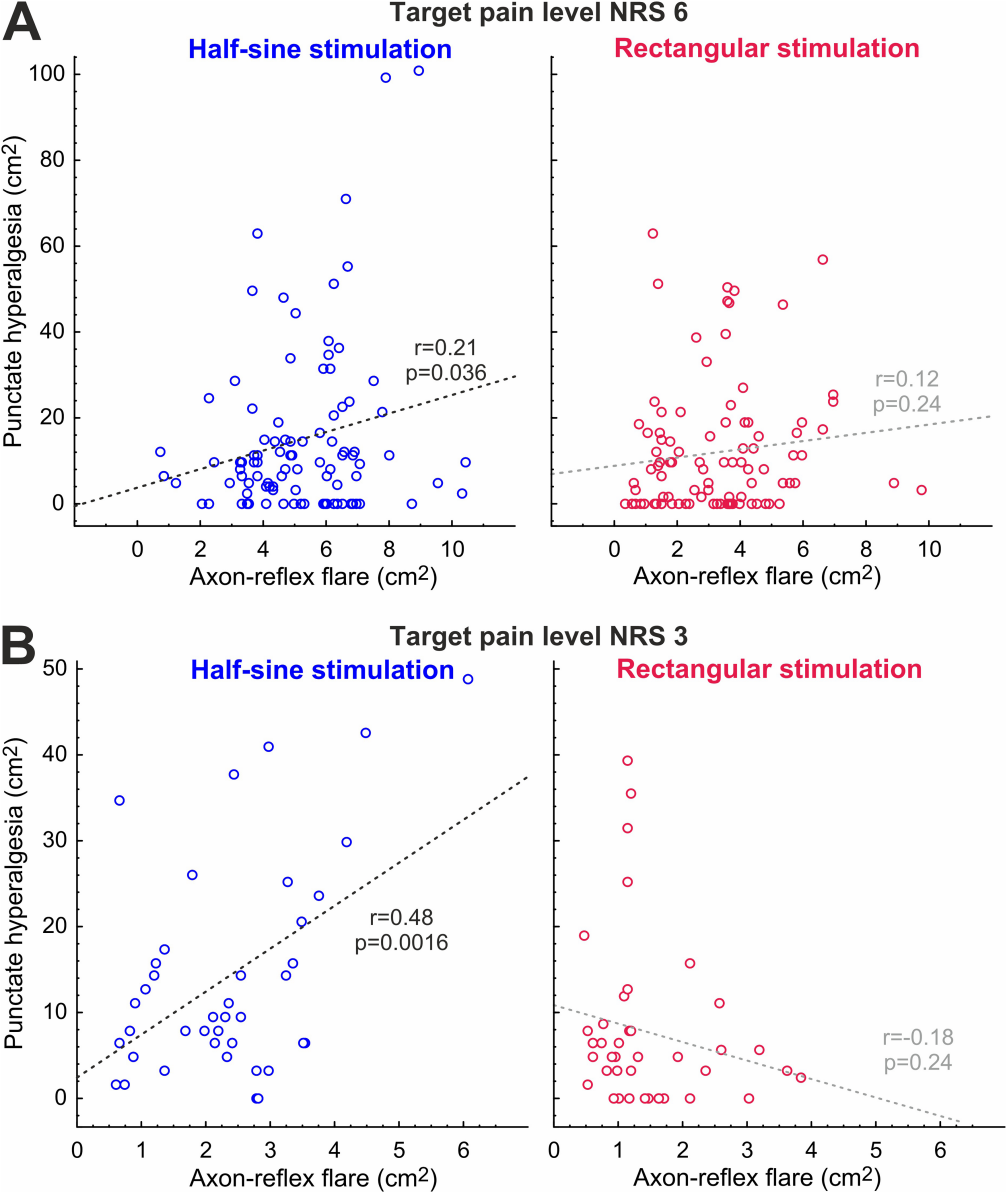
Correlation between development of axon-reflex flare and hyperalgesia at NRS 6 **(A)** and NRS 3 **(B)** for half-sine and rectangular stimulation; *r*, Pearson’s *r*.

When plotting the flare areas against areas of secondary hyperalgesia assessed during the first 35 min of stimulation, there was a significant correlation for HS stimulation at NRS 3 (*r* = 0.48, *p* = 0.0016; Figure 7B), and this correlation remained significant at NRS 6 (*r* = 0.21, *p* = 0.036) (Figure 7A). Flare and hyperalgesia were found to be completely unrelated for R stimulation at NRS 3 (*r* = 0.18, *p* = 0.24) and at NRS 6 (*r* = 0.12, *p* = 0.24) (Figure 7).

## 4 Discussion

While C-fiber-optimized electrical stimulation facilitated the generation of the axon reflex erythema, it did not increase the area of secondary hyperalgesia. Secondary mechanical hyperalgesia developed earlier as compared to traditional rectangular stimulation and correlated with the axon reflex area. Accordingly, this correlation was not observed with conventional rectangular stimulation. Most importantly, the final area of punctate hyperalgesia did not differ between the two electrical stimulation profiles. Our data therefore suggest that nociceptive fibers inducing the axon-reflex flare may contribute to the development of secondary mechanical hyperalgesia, but they are not crucial for its induction. Probably, an additional fiber class that is not involved in the peripheral flare response contributes to the development of secondary mechanical hyperalgesia.

### 4.1 Evidence for preferred activation of C-nociceptors by 25 ms half-sine stimulation: pain and axon reflex flare response

While detection thresholds did not differ significantly between the two electrical stimulation profiles, our data confirm that the current intensities required for pain thresholds were lower for the half-sine stimulation as previously reported rectangular stimuli (27). This result was expected based on the different expressions of voltage-sensitive sodium channels (NaVs) in A- and C-fibers. The short rectangular pulses are ideal to activate NaV1.6 on A-fibers whereas the slower depolarization induced by sinusoidal pulses facilitates its closed-state inactivation, and thus, myelinated fibers are less sensitive to such stimuli. In contrast, NaV1.7 and NaV1.8 in C-fibers (28, 29) are less prone to closed-state inactivation facilitating C-fiber activation upon slow depolarization (9, 13). Moreover, based on the long chronaxie of unmyelinated fibers (16), pulses of longer duration are more efficient. For extracellular stimulation, the axonal membrane potential is proportional to the second temporal derivative of the electrical stimulus (30), and thus sinusoidal rather than long-lasting rectangular pulses were used.

Indeed, axon-reflex flare responses that have been shown to depend on the activation of mechano-insensitive “silent” nociceptors (4) were induced preferentially by half-sine stimulation and were evident even at stimulus intensities as low as the detection threshold. Rectangular pulses, even at the pain threshold, did not induce a significant flare response (Figure 4). Our data are in line with the hypothesis that half-sine electrical stimulation more selectively activates “silent” nociceptors as compared to rectangular stimulation. However, upon increasing the stimulus intensity of the rectangular pulse, we expect that not only A-nociceptors but also some silent C-nociceptors will be activated. We therefore also investigated suprathreshold pain responses inducing a pain level of 3/10. In fact, at this suprathreshold stimulation level, rectangular pulses induced a small axon reflex flare response comparable in size to the one seen after half-sine stimulation at the detection threshold, but with >3 times smaller diameter as compared to half-sine stimulation at the same pain level.

### 4.2 Arguments for the link between silent nociceptors and secondary mechanical hyperalgesia

Long-lasting chemical responses to capsaicin injection in human silent nociceptors, but not in polymodal nociceptors, have been suggested as an argument for the crucial role of silent nociceptors for the induction of secondary mechanical hyperalgesia (31). Moreover, the area of secondary hyperalgesia is known to depend on the level of nociceptive input as has been shown during peripheral nerve fiber conduction block (11) and in clinical pain conditions (27). Even in the same electrical pain model, it has been shown that the reduction of nociceptor excitability by systemic administration of lidocaine reduced both the spatial extent of the peripheral axon-reflex flare and the area of secondary hyperalgesia (1), which would speak in favor of a link. Therefore, we expected that increased spinal input via “silent” nociceptors would also lead to larger areas of secondary hyperalgesia. However, our results support this link only for the initial assessment (Figure 6, 5 min) and only for the low stimulus intensity (NRS 3) for which we observed a larger area of punctate hyperalgesia for the half-sine stimulation. Beyond this very limited direct evidence, we found a positive correlation between the areas of flare response and secondary mechanical hyperalgesia only for the half-sine stimulation and for both stimulus intensities (Figure 7). Although this effect appears to be stronger, such a correlation can only be considered indirect evidence for the involvement of silent nociceptors in the induction of punctate hyperalgesia and should not be overestimated.

### 4.3 Arguments against the link between silent nociceptors and secondary mechanical hyperalgesia

If the development of secondary hyperalgesia were exclusively dependent on the activation of “silent” C-nociceptors, we would have expected an increase in both flare response and area of secondary hyperalgesia for half-sine vs. rectangular profile. However, while the difference in flare responses between the stimulation profiles was very clear, the area under the curve and maximum hyperalgesic areas did not significantly differ. Thus, our results clearly speak against silent nociceptors being indispensable for the induction of secondary mechanical hyperalgesia.

## 5 Possible explanations

One might argue that the induction of central sensitization might be peculiar requiring only a few action potentials to develop (32) and thereby leading to a saturation phenomenon or ceiling effect (33). Thus, suprathreshold rectangular stimulation might have activated some “silent” nociceptors, which are too few to evoke a stable axon-reflex flare response compared to half-sine stimuli, but whose spinal input could induce the same area of secondary hyperalgesia over time. However, a clear dose response for capsaicin-induced punctate hyperalgesia and flare has been found that was virtually linear between 0.1 and 100 µg of capsaicin (34) including the range of pain ratings reported here. Therefore, saturation or ceiling effects are highly improbable explanations for our results.

Given the development of secondary hyperalgesia without axon reflex following rectangular stimulation (Figure 6), our results likely indicate that “silent” nociceptor activation induces both peripheral axon-reflex flare responses and central sensitization but that there could be other nociceptor classes capable of inducing central sensitization without necessarily eliciting the axon-reflex flare. A potential example in the literature is prolonged non-painful warming of human skin that induced secondary hyperalgesia in most subjects without a concomitant flare response (35). However, injection of hypertonic saline into deep somatic tissue (interspinous ligament) provokes secondary hyperalgesia without flare response in the skin (36). Teleologically, the depth aspect appears to be of importance: secondary mechanical hyperalgesia induced by nociceptors innervating deeper tissues would make sense, as protection appears to be the more adequate response as compared to withdrawal. Accordingly, as suggested decades ago by Sir Thomas Lewis (37), such a “nocifensor system” can be activated by deep tissue injury contributing to the spreading of hyperalgesia. Unfortunately, our knowledge about nociceptor classes of deeper skin layers is limited, but we have anecdotal evidence of subcutaneous C-nociceptors in human microneurography only activated by strong pinching of the skin (Torebjörk, Handwerker, Schmelz, personal communication) and also recorded from such subcutaneous nociceptors in pig (Rukwied, Schmelz, in preparation). The deep location of such nociceptors would limit their ability to provoke a neurogenic flare in the dermis.

### 5.1 Strengths and limitations

The blinding of participants to electrical stimulation represented a strength of the present study. In addition, the consistency between the results of the rectangular stimulation profile in the present study with those of the previous study of our group underlines the quality of our data. However, this study is limited by the small sample size for the assessments at pain level NRS 3 and by the unpowered secondary outcomes. Furthermore, the interpretation of allodynia and its comparison between the two stimulation profiles was limited using the investigated model.

## 6 Conclusion

Albeit slow depolarizing half-sine stimulation at low stimulation levels triggers hyperalgesia faster and a positive correlation between hyperalgesia and flare was observed for this electrical stimulation profile, rectangular stimulation also provoked hyperalgesia but without concomitant flare development indicating that “silent” nociceptors are not the only pathway of noxious peripheral input to induce central sensitization. Growing knowledge in expression patterns of single nociceptors in humans will potentially help to identify possible candidates contributing to secondary hyperalgesia.

## Data Availability

The raw data supporting the conclusions of this article will be made available by the authors, without undue reservation.

## Supplementary material

Supplementary Figure S1

The flux image is generated by a total of 230 lines. The white line (1) marks the line before stimulation while the red line (2) marks the end of stimulation. The warmer colors represent a higher blood flow (interpreted as axon-reflex flare), as shown here above the red line.

Supplementary Figure S2

Line scan during electrical stimulation with rectangular pulses. The brighter colors represent a higher blood flow (interpreted as axon-reflex flare). During stimulation no blood flow changes was observed.

Supplementary Figure S3

Line scan during electrical stimulation with half-sine pulses after image correction. The white line (1) marks the beginning of stimulation. The calculated mean of the three longest lines (2, 3, and 4) represent the baseline length without stimulation. The brighter colors represent a higher blood flow (interpreted as axon-reflex flare).

Supplementary Figure S4

Line scan during electrical stimulation with halfsine pulses. The white line (1) marks the beginning of stimulation. The calculated mean of the three longest lines (2, 3 and 4) represents the expansion of blood flow increase during respectively after stimulation. The brighter colors represent a higher blood flow (interpreted as axon-reflex flare).

Supplementary Figure S5

Flare square recorded during electrical stimulation with half-sine pulses. The brighter colors represent a higher blood flow (interpreted as axon-reflex flare). 1 = 0 min, 2 = 5 min, 3 = 15 min, 4 = 25 min, 5 = 35 min, 6 = 45 min, 7 = 55 min, 8 = 65 min.

Supplementary Figure S6

Flare square after image correction recorded during electrical stimulation with half-sine pulses. The brighter colors represent a higher blood flow (interpreted as axon-reflex flare). 1 = 0 min, 2 = 5 min, 3 = 15 min, 4 = 25 min, 5 = 35 min, 6 = 45 min, 7 = 55 min, 8 = 65 min.

## References

[1] Koppert W Dern SK Sittl R Albrecht S Schuttler J Schmelz M. A new model of electrically evoked pain and hyperalgesia in human skin: the effects of intravenous alfentanil, S(+)-ketamine, and lidocaine. Anesthesiology. (2001) 95(2):395–402. 10.1097/00000542-200108000-00022 11506112

[2] Bandschapp O Filitz J Ihmsen H Berset A Urwyler A Koppert W Analgesic and antihyperalgesic properties of propofol in a human pain model. Anesthesiology. (2010) 113(2):421–8. 10.1097/ALN.0b013e3181e33ac8 20613472

[3] Weidner C Schmelz M Schmidt R Hansson B Handwerker HO Torebjork HE. Functional attributes discriminating mechano-insensitive and mechano-responsive C nociceptors in human skin. J Neurosci. (1999) 19(22):10184–90. 10.1523/JNEUROSCI.19-22-10184.1999 10559426 PMC6782981

[4] Schmelz M Michael K Weidner C Schmidt R Torebjork HE Handwerker HO. Which nerve fibers mediate the axon reflex flare in human skin? Neuroreport. (2000) 11(3):645–8. 10.1097/00001756-200002280-00041 10718329

[5] Dusch M Schley M Rukwied R Schmelz M. Rapid flare development evoked by current frequency-dependent stimulation analyzed by full-field laser perfusion imaging. Neuroreport. (2007) 18(11):1101–5. 10.1097/WNR.0b013e3281e72cff 17589307

[6] Kleggetveit IP Namer B Schmidt R Helas T Ruckel M Orstavik K High spontaneous activity of C-nociceptors in painful polyneuropathy. Pain. (2012) 153(10):2040–7. 10.1016/j.pain.2012.05.017 22986070

[7] Orstavik K Weidner C Schmidt R Schmelz M Hilliges M Jorum E Pathological C-fibres in patients with a chronic painful condition. Brain. (2003) 126(Pt 3):567–78. 10.1093/brain/awg060 12566278

[8] Serra J Bostock H Sola R Aleu J Garcia E Cokic B Microneurographic identification of spontaneous activity in C-nociceptors in neuropathic pain states in humans and rats. Pain. (2012) 153(1):42–55. 10.1016/j.pain.2011.08.015 21993185

[9] Jonas R Namer B Stockinger L Chisholm K Schnakenberg M Landmann G Tuning in C-nociceptors to reveal mechanisms in chronic neuropathic pain. Ann Neurol. (2018) 83(5):945–57. 10.1002/ana.25231 29659054

[10] LaMotte RH Shain CN Simone DA Tsai E-FP. Neurogenic hyperalgesia: psychophysical studies of underlying mechanisms. J Neurophysiol. (1991) 66(1):190–211. 10.1152/jn.1991.66.1.190 1919666

[11] LaMotte RH Lundberg LE Torebjörk HE. Pain, hyperalgesia and activity in nociceptive-C units in humans after intradermal injection of capsaicin. J Physiol. (1992) 448:749–64. 10.1113/jphysiol.1992.sp019068 1593488 PMC1176226

[12] Schmidt R Schmelz M Forster C Ringkamp M Torebjörk HE Handwerker HO. Novel classes of responsive and unresponsive C nociceptors in human skin. J Neurosci. (1995) 15(1):333–41. 10.1523/JNEUROSCI.15-01-00333.1995 7823139 PMC6578337

[13] Rukwied R Thomas C Obreja O Werland F Kleggetveit IP Jorum E Slow depolarizing stimuli differentially activate mechanosensitive and silent C nociceptors in human and pig skin. Pain. (2020) 161(9):2119–28. 10.1097/j.pain.0000000000001912 32379219

[14] Jonas R Namer B Schnakenberg M Soares S Pakalniskis J Carr R Sympathetic efferent neurons are less sensitive than nociceptors to 4 Hz sinusoidal stimulation. Eur J Pain. (2020) 24(1):122–33. 10.1002/ejp.1467 31392805

[15] Tigerholm J Hoberg TN Bronnum D Vittinghus M Frahm KS Morch CD. Small and large cutaneous fibers display different excitability properties to slowly increasing ramp pulses. J Neurophysiol. (2020) 124(3):883–94. 10.1152/jn.00629.2019 32783585

[16] Schneider T Filip J Soares S Sohns K Carr R Rukwied R Optimized electrical stimulation of C-nociceptors in humans based on the chronaxie of porcine C-fibers. J Pain. (2023) 24(6):957–69. 10.1016/j.jpain.2023.01.009 36681314

[17] Schneider T Zurbriggen L Dieterle M Mauermann E Frei P Mercer-Chalmers-Bender K Pain response to cannabidiol in induced acute nociceptive pain, allodynia, and hyperalgesia by using a model mimicking acute pain in healthy adults in a randomized trial (CANAB I). Pain. (2022) 163(1):e62–71. 10.1097/j.pain.0000000000002310 34086631

[18] Dieterle M Zurbriggen L Mauermann E Mercer-Chalmers-Bender K Frei P Ruppen W Pain response to cannabidiol in opioid-induced hyperalgesia, acute nociceptive pain and allodynia by using a model mimicking acute pain in healthy adults in a randomized trial (CANAB II). Pain. (2022) 163(10):1919–28. 10.1097/j.pain.0000000000002591 35239547 PMC9982727

[19] Mauermann E Filitz J Dolder P Rentsch KM Bandschapp O Ruppen W. Does fentanyl lead to opioid-induced hyperalgesia in healthy volunteers?: a double-blind, randomized, crossover trial. Anesthesiology. (2016) 124(2):453–63. 10.1097/ALN.0000000000000976 26655493

[20] Koppert W Sittl R Scheuber K Alsheimer M Schmelz M Schuttler J. Differential modulation of remifentanil-induced analgesia and postinfusion hyperalgesia by S-ketamine and clonidine in humans. Anesthesiology. (2003) 99(1):152–9. 10.1097/00000542-200307000-00025 12826855

[21] Schnakenberg M Thomas C Schmelz M Rukwied R. Nerve growth factor sensitizes nociceptors to C-fibre selective supra-threshold electrical stimuli in human skin. Eur J Pain. (2021) 25(2):385–97. 10.1002/ejp.1678 33064901

[22] Chizh BA O'Donnell MB Napolitano A Wang J Brooke AC Aylott MC The effects of the TRPV1 antagonist SB-705498 on TRPV1 receptor-mediated activity and inflammatory hyperalgesia in humans. Pain. (2007) 132(1–2):132–41. 10.1016/j.pain.2007.06.006 17659837

[23] Geber C Fondel R Kramer HH Rolke R Treede RD Sommer C Psychophysics, flare, and neurosecretory function in human pain models: capsaicin versus electrically evoked pain. J Pain. (2007) 8(6):503–14. 10.1016/j.jpain.2007.01.008 17434803

[24] Cepeda MS Africano JM Polo R Alcala R Carr DB. What decline in pain intensity is meaningful to patients with acute pain? Pain. (2003) 105(1–2):151–7. 10.1016/S0304-3959(03)00176-3 14499431

[25] Blanca MJ Arnau J Garcia-Castro FJ Alarcon R Bono R. Non-normal data in repeated measures ANOVA: impact on type I error and power. Psicothema. (2023) 35(1):21–9. 10.7334/psicothema2022.292 36695847

[26] Shuster JJ. Student t-tests for potentially abnormal data. Stat Med. (2009) 28(16):2170–84. 10.1002/sim.3581 19326398 PMC3666168

[27] Koltzenburg M Torebjörk HE Wahren LK. Nociceptor modulated central sensitization causes mechanical hyperalgesia in acute chemogenic and chronic neuropathic pain. Brain. (1994) 117(Pt 3):579–91. 10.1093/brain/117.3.579 8032867

[28] Petersson ME Obreja O Lampert A Carr RW Schmelz M Fransen E. Differential axonal conduction patterns of mechano-sensitive and mechano-insensitive nociceptors–a combined experimental and modelling study. PLoS One. (2014) 9(8):e103556. 10.1371/journal.pone.0103556 25136824 PMC4138079

[29] Tigerholm J Petersson ME Obreja O Eberhardt E Namer B Weidner C C-Fiber recovery cycle supernormality depends on ion concentration and ion channel permeability. Biophys J. (2015) 108(5):1057–71. 10.1016/j.bpj.2014.12.034 25762318 PMC4816283

[30] Spach MS Barr RC Serwer GA Kootsey JM Johnson EA. Extracellular potentials related to intracellular action potentials in the dog Purkinje system. Circ Res. (1972) 30(5):505–19. 10.1161/01.RES.30.5.505 5026754

[31] Schmelz M Schmid R Handwerker HO Torebjork HE. Encoding of burning pain from capsaicin-treated human skin in two categories of unmyelinated nerve fibres. Brain. (2000) 123 (Pt 3):560–71. 10.1093/brain/123.3.560 10686178

[32] Sauerstein K Liebelt J Namer B Schmidt R Rukwied R Schmelz M. Low-frequency stimulation of silent nociceptors induces secondary mechanical hyperalgesia in human skin. Neuroscience. (2018) 387:4–12. 10.1016/j.neuroscience.2018.03.006 29551562

[33] Huang JH Ali Z Travison TG Campbell JN Meyer RA. Spatial mapping of the zone of secondary hyperalgesia reveals a gradual decline of pain with distance but sharp borders. Pain. (2000) 86(1–2):33–42. 10.1016/S0304-3959(99)00314-0 10779658

[34] Simone DA Baumann TK LaMotte RH. Dose-dependent pain and mechanical hyperalgesia in humans after intradermal injection of capsaicin. Pain. (1989) 38(1):99–107. 10.1016/0304-3959(89)90079-1 2780068

[35] Cervero F Gilbert R Hammond RGE Tanner J. Development of secondary hyperalgesia following nonpainful thermal stimulation of the skin a psychophysical study in man. Pain. (1993) 54:181–9. 10.1016/0304-3959(93)90207-6 8233532

[36] Tsao H Tucker KJ Coppieters MW Hodges PW. Experimentally induced low back pain from hypertonic saline injections into lumbar interspinous ligament and erector spinae muscle. Pain. (2010) 150(1):167–72. 10.1016/j.pain.2010.04.023 20510516

[37] Lewis T. Nocifensor system of nerves. Br Med J. (1937) 1(3973):431–5. 10.1136/bmj.1.3973.431 20780499 PMC2088263

