# Supplementary figures and images for: Preferential C-nociceptor stimulation facilitates peripheral axon reflex flare, but not secondary mechanical hyperalgesia
Source: Front Pain Res (Lausanne). 2025 Mar 19;6:1556429. doi: 10.3389/fpain.2025.1556429 (PMC11961941; doi:10.3389/fpain.2025.1556429)

## Supplements

### Supplement 1

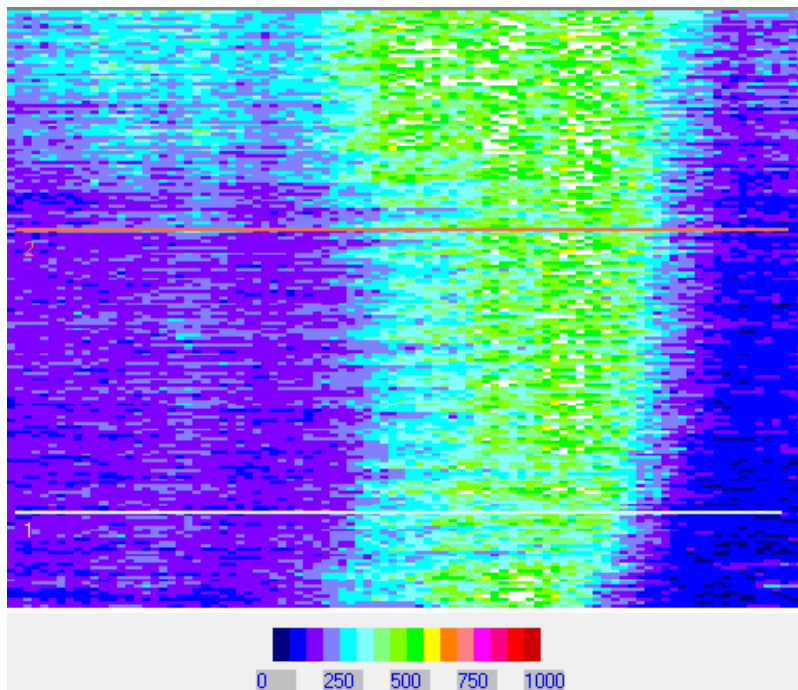

### Supplement 2

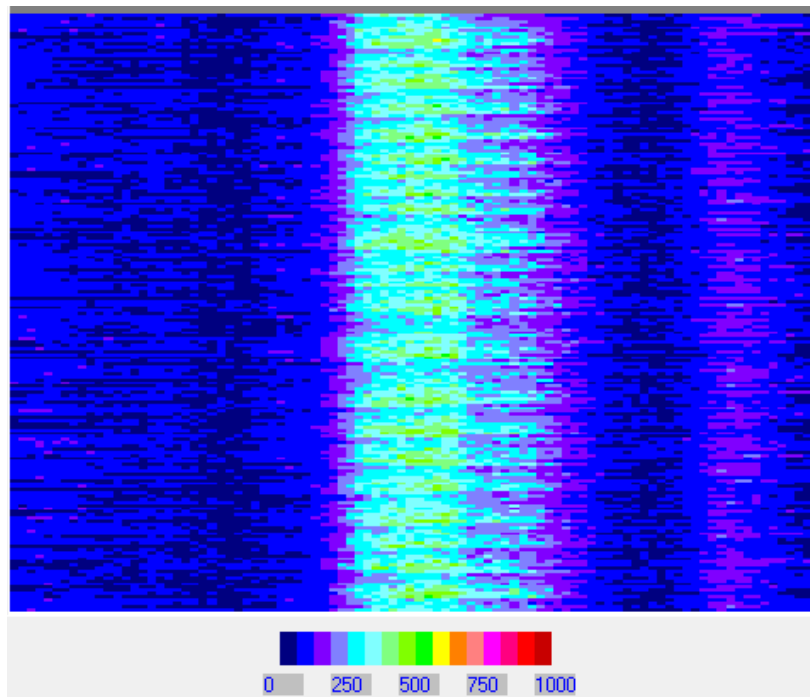

### Supplement 3

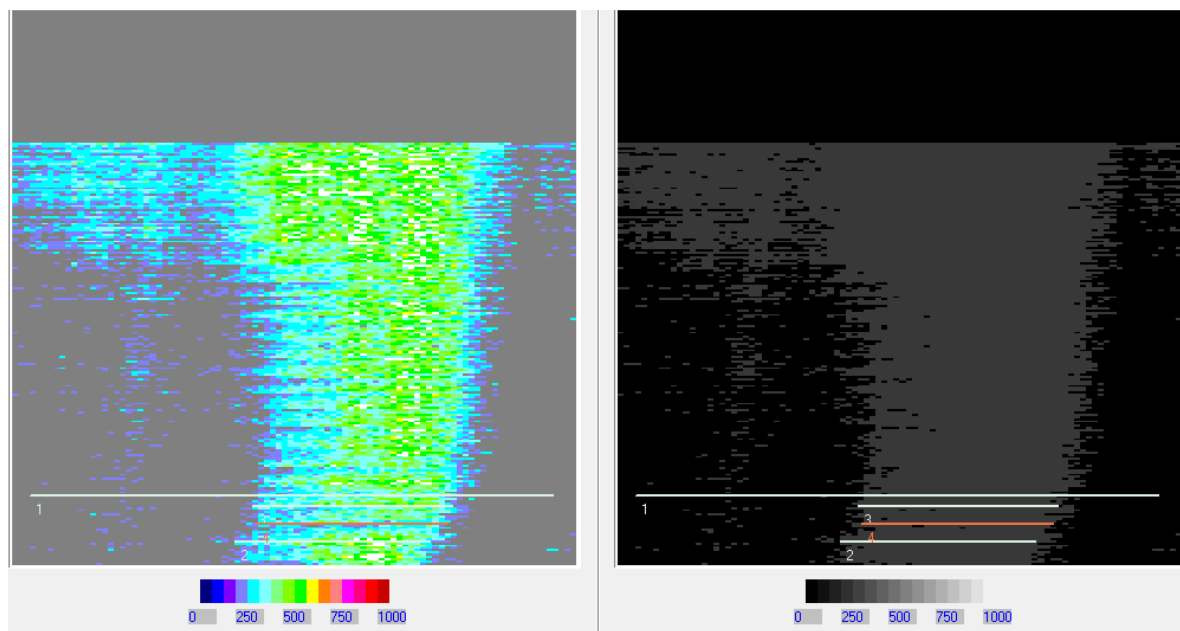

### Supplement 4

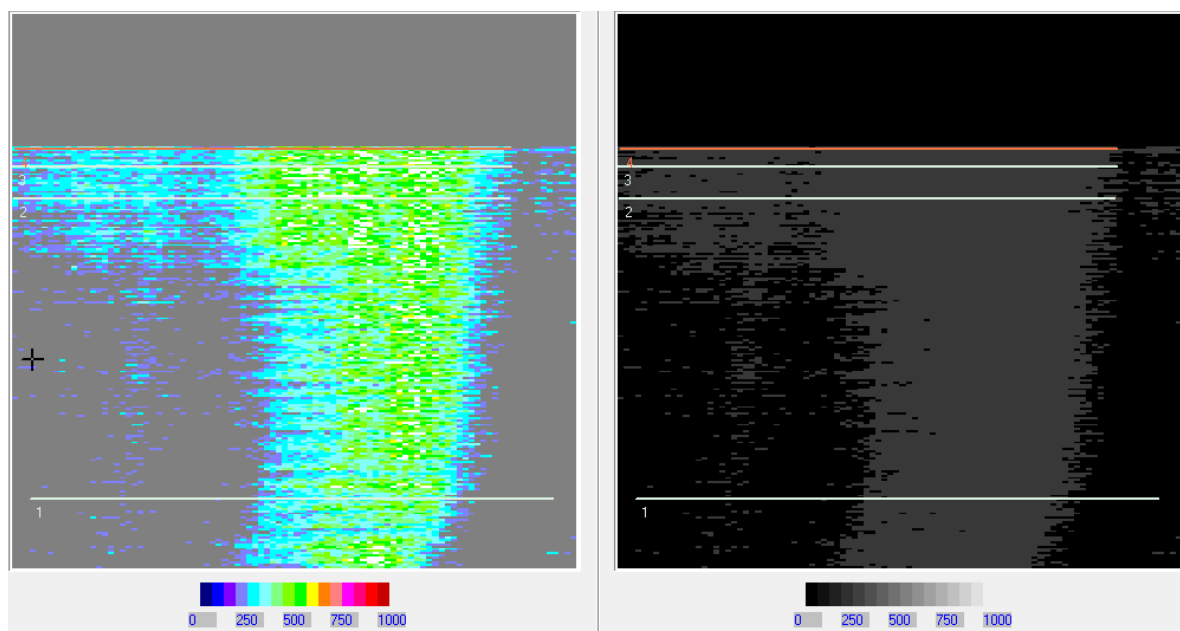

## Supplement 5

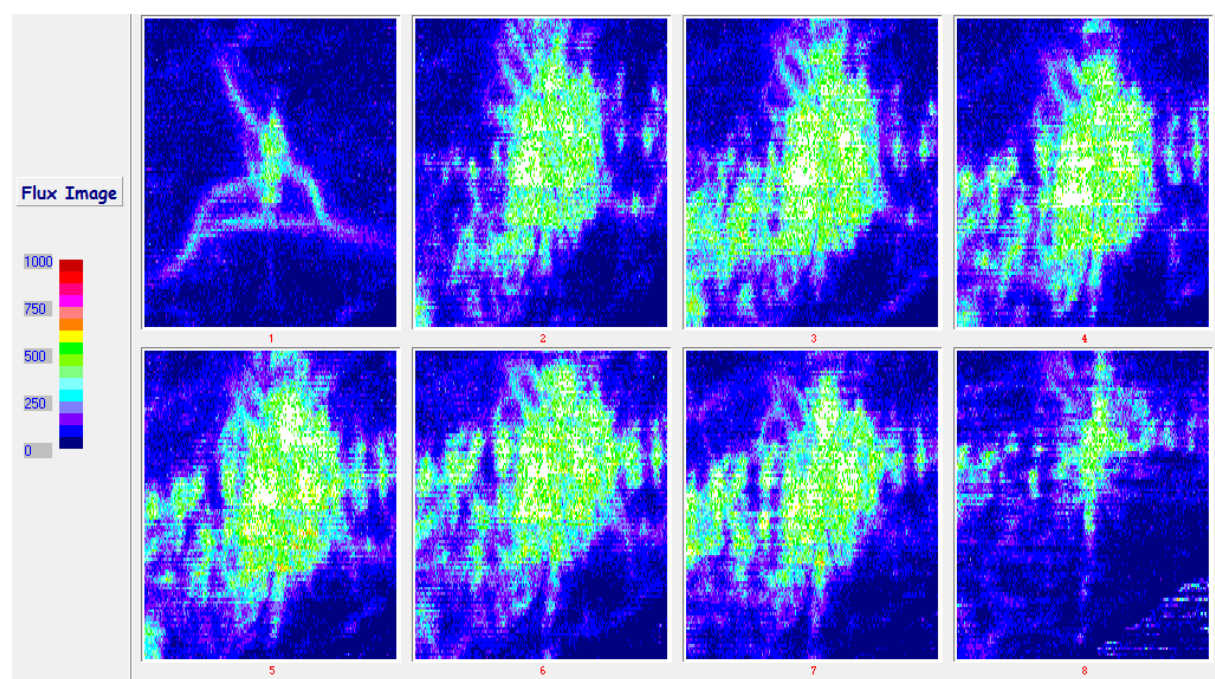

## Supplement 6

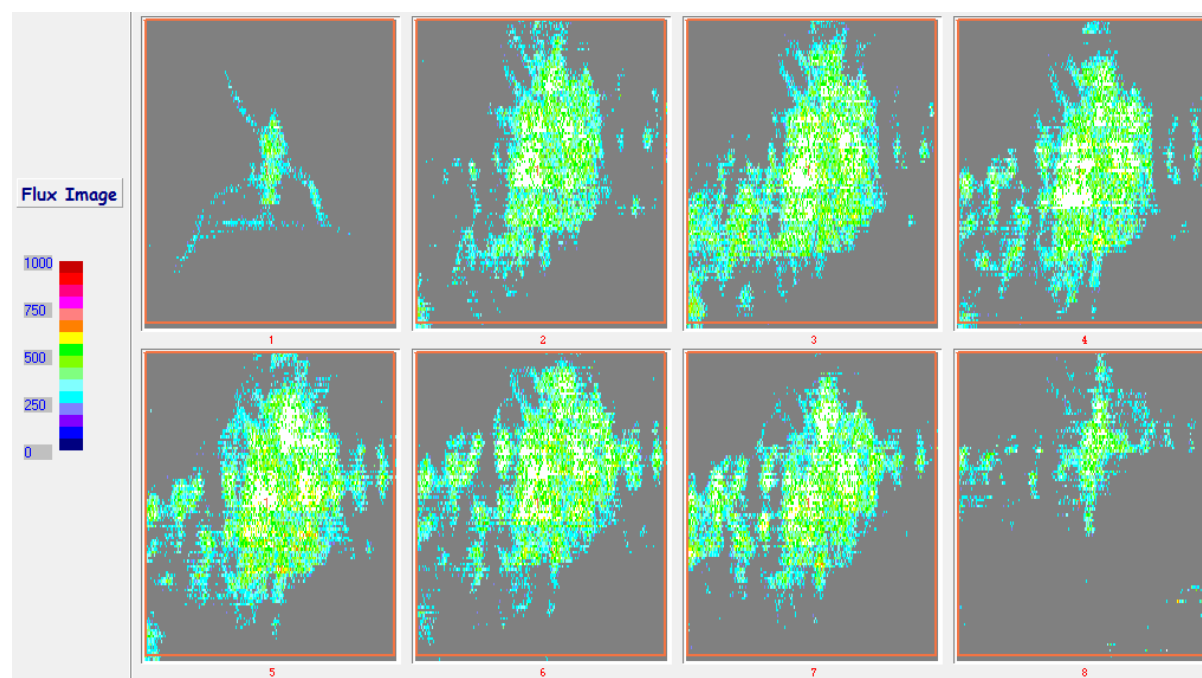

Supplement: Supplementary file 1 [file Datasheet1.pdf]
